# Simple Method to Measure the Aerodynamic Size Distribution of Porous Particles Generated on Lyophilizate for Dry Powder Inhalation

**DOI:** 10.3390/pharmaceutics12100976

**Published:** 2020-10-15

**Authors:** Kahori Miyamoto, Hiroaki Taga, Tomomi Akita, Chikamasa Yamashita

**Affiliations:** Department of Pharmaceutics and Drug Delivery, Faculty of Pharmaceutical Sciences, Tokyo University of Science, 2641 Yamazaki, Noda, Chiba 278-8510, Japan; 3A17706@ed.tus.ac.jp (K.M.); j3a11051@ed.tus.ac.jp (H.T.); akitat@rs.tus.ac.jp (T.A.)

**Keywords:** dry powder inhalation, aerodynamic particle size distribution, time-of-flight measurement, porous particles

## Abstract

Recently, statistical techniques such as design of experiments are being applied for efficient optimization of oral formulations. To use these statistical techniques for inhalation formulations, efficient methods for rapid determination of the aerodynamic particle size distribution of many samples are needed. Therefore, we aimed to develop a simple method to measure aerodynamic particle size distribution that closely agrees with the results of inhalation characteristic tests. We added attachments for dispersion to the aerodynamic particle sizer (APS) so that formulations could be dispersed under the same condition as for multi-stage liquid impinger (MSLI) measurement. Then, we examined the correlation between MSLI and APS using lyophilizate for dry powder inhalation formulations that generate porous particles just on inhalation. It is difficult to obtain the accurate aerodynamic particle size distribution of porous particles by APS because the particle density is difficult to estimate accurately. However, there was a significant correlation between MSLI and APS when the particle density settings for APS measurement was calculated by a conversion factor based on the result of MSLI. The APS with dispersion attachments and this conversion factor can measure a number of samples in a short time, thereby enabling more efficient optimization of dry powder inhalers.

## 1. Introduction

**Figure 1:**
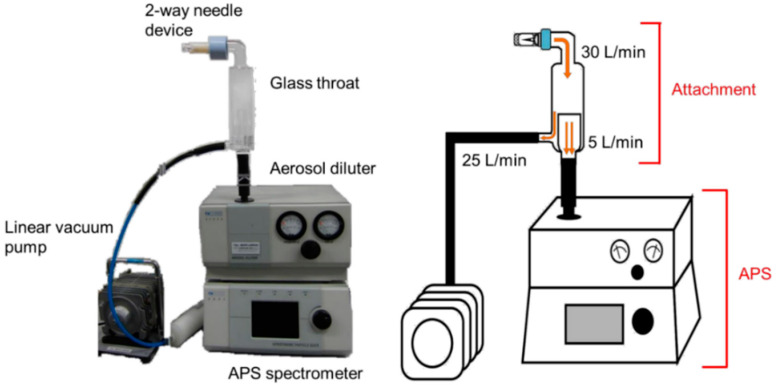
The aerodynamic particle sizer (APS) system with dispersion attachments. Grass throat and linear vacuum pump were added so that formulations can be dispersed under the same condition as for multi-stage liquid impinger measurement (flow rate of 30 L/min at a pressure drop of 4 kPa with two-way needle device). Dispersed particles are measured at flow rate of 5 L/min, which is the standard flow rate for APS measurement.

**Figure 2:**
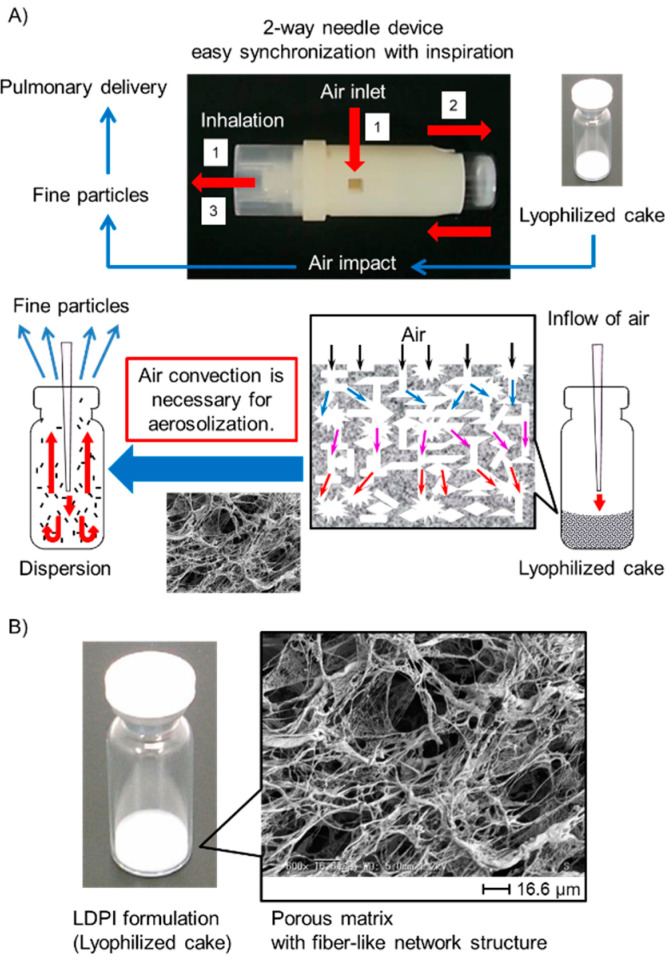
(**A**) Mechanism of lyophilizate for dry powder inhalation (LDPI) system. (1) Air is introduced into the vial in synchronization with the patient’s inhalation through the two-way needle device. (2) Lyophilizate with a porous matrix structure is broken into pieces by air impact and aerosolized by the convection flow of air in the vial. (3) Porous particles reconstructed from pieces of lyophilizate are emitted through the device. (**B**) Enlarged picture of LDPI formulation and scanning electron microscope image. LDPI formulation is a lyophilized cake having porous matrix with fiber-like network structure.

Currently available inhaled drug delivery systems for topical administration to the lung can be divided into three principal categories: nebulizers, pressurized metered-dose inhalers, and dry powder inhalers (DPIs). Among these, DPIs have recently attracted attention due to their convenience and because of environmental considerations [1,2]. In the DPI formulations, aerosols need to have aerodynamic diameters between 0.5 and 5 µm to deliver the drug to the lungs [3,4]. The aerodynamic diameter is defined by Equation (1) [5].

(1) $$\mathrm{Aerodynamic}\;\mathrm{diameter}=\mathrm{Geometric}\;\mathrm{diameter}\;\times \sqrt{\frac{\rho_{P}}{\rho_{0}\chi }}\;,$$

where *ρ*_p_ and *ρ*_0_ are the particle and unit densities, respectively, and *χ* is the dynamic shape factor. Aerosol particle design therefore involves two basic strategies. Either particles are made with standard density with geometric diameters between 0.5 and 5 µm, or they are created with a non-standard density with aerodynamic diameters between 0.5 and 5 µm in contrast to geometric diameters outside the standard range. Conventional DPIs use the first strategy, whereas large porous particles provide an example of the second strategy [6,7]. Large porous particles are recently becoming popular as the technique for both local and systemic applications by the pulmonary route to the lungs [8,9,10,11,12]. A major advantage of large porous particles compared to conventional DPIs is their aerosolization performance [13,14,15]. Whichever of the above two strategies is selected, measurement of the aerodynamic particle size distribution is essential for optimization of DPIs [16,17]. Recently, statistical techniques such as design of experiments and response surface methodology are being applied for efficient optimization of oral formulations [18,19,20]. DPIs are also preferred to be optimized efficiently by these techniques. For efficient optimization of DPIs by statistical techniques, an efficient method for rapid determination of the aerodynamic particle size distribution of many samples is needed. However, inhalation characteristic tests by pharmacopeial methods (e.g., cascade impactor and multi-stage liquid impinger (MSLI)) require much time even for only a few samples [21]. From this point, pharmacopeial methods are not preferable for optimization by statistical techniques [22]. Time-of-flight (TOF) measurement is one method that can measure the aerodynamic particle size distribution of many samples in a short time [21,23]. TOF measurement is easy to perform, but the settings of particle density greatly affect the results of measurement [24,25,26]. To obtain accurate aerodynamic particle size distribution by TOF measurement, the particle density needs to be calculated accurately. However, it is difficult to calculate the accurate particle density of DPIs formulated with large porous particles because large porous particles generally behave as agglomerates of primary particles. Thus, we aimed to develop a simple method based on TOF theory to measure the aerodynamic particle size distribution that closely agrees with the results of inhalation characteristic tests by the pharmacopeial method of MSLI even in the case of large porous particles. First, we added attachments for dispersion to the aerodynamic particle sizer (APS), as shown in Figure 1, so that particles dispersed from a dosage form under the same condition as for MSLI measurement could be measured by APS. Then, we examined the correlation between MSLI and the APS with dispersion attachments using lyophilizate for dry powder inhalation (LDPI) formulations [27,28,29,30,31] as formulations with porous particles. In the LDPI system, porous particles suitable for pulmonary administration are generated on inhalation. As shown in Figure 2, one characteristic of LDPI is that a formulation consists not of particles but of a freeze-dried cake with a porous matrix structure that is constructed by sublimation of water during lyophilization. This porous matrix is broken into pieces because of the convection flow of air introduced in synchronization with the patient’s inspiration as with jet milling (Video S1). Then, these pieces are reconstructed into porous particles suitable for pulmonary administration in the vial, followed by emission of the porous particles through the LDPI device. Thus, LDPI can effectively deliver drugs to lungs as porous particles generated from the freeze-dried cake upon inhalation.

The aim of this study was to develop a measurement method of the aerodynamic particle size distribution which can be used in optimization of DPIs by statistical techniques instead of pharmacopeial methods. Requirements were that many samples can be measured in a short time and that the results of pharmacopeial methods can be estimated from the result of the developed method. For rapid measurement, we used APS. To obtain the results from which the results of pharmacopeial method can be estimated, we adjusted the dispersion condition and contrived to calculate the particle density for APS measurement.

## 2. Materials and Methods

### 2.1. Materials

hGhrelin was obtained from Asubio Pharma (Kobe, Japan). Tamibarotene (Am80) was provided by Ituu Laboratory (Tokyo, Japan). Nicotinamide (VB_3_) was purchased from Sigma-Aldrich Japan (Tokyo, Japan). Cyanocobalamin (VB_12_) and polyoxyethylene sorbitan monooleate (Tween 80) were purchased from Tokyo Chemical Industry (Tokyo, Japan). Ammonium acetate (reagent grade) was purchased from Nacalai Tesque (Kyoto, Japan). L-phenylalanine (Phe, special grade), ethanol (99.5%) (reagent grade), acetonitrile (HPLC (high-performance liquid chromatography) grade), benzalkonium chloride, acetic acid (special grade), dimethyl sulfoxide (DMSO, reagent grade), trifluoro acetic acid (TFA, special grade), and methanol (HPLC grade) were all purchased from Fujifilm Wako Pure Chemical Industries (Osaka, Japan).

The packaging materials used in this study were obtained from the following commercial vendors: 2-mL VIST glass vials from Daiwa Special Glass (Osaka, Japan) and rubber stoppers (F5-43) from Sumitomo Rubber Industries (Kobe, Japan).

### 2.2. Preparation of Freeze-Dried Cake

Model formulations for LDPI were prepared by using the following materials as main drugs: hGhrelin, VB_3_, VB_12_, and Am80. hGhrelin was selected as a representative water-soluble peptide, and VB_3_ and VB_12_ were selected as representative water-soluble compounds. Am80 was selected as a representative hydrophobic compound. Am80 is a curative agent for alveolar destruction in chronic obstructive pulmonary disease (COPD). We previously showed that pulmonary administration of Am80 repaired alveoli in COPD model mice [32,33].

Stock solutions of Phe were prepared by dissolving them in purified water (20 mg/mL), and those of the water-soluble main drugs (hGhrelin, VB_3_, and VB_12_) were prepared by dissolving them in purified water (2 mg/mL). Stock solutions (Table S1) were mixed and diluted by purified water to obtain the target concentration (hGhrelin and VB_3_ 0.2 mg/mL and Phe 1.0 mg/mL; VB_12_ 0.2 mg/mL and Phe 1.2 mg/mL; Phe 0.4 mg/mL). Five hundred microliters of the solution were filled in a vial and lyophilized by a benchtop freeze dryer (FreeZone Triad 7400030, LABCONCO, Kansas City, MO, USA). Am80 was dissolved in ethanol (99.5%) (20 mg/mL) and then suspended in diluted stock solutions of Phe to obtain the target concentration (Am80 and Phe 0.2 mg/mL, respectively). Five hundred microliters of the suspension were filled in a vial and frozen using liquid nitrogen followed by lyophilization by a benchtop freeze dryer. The lyophilization conditions are as follows. Shelf cooling was performed from room temperature to −50 °C (≤0.5 °C/min), and shelf temperature was held at −50 °C for 4 h. Primary drying was performed at a shelf temperature of −30 °C and pressure of 1.0 Pa for 11 h. In the secondary drying step, shelf temperature was ramped to 35 °C (0.17 °C/min) for 5 h and then decreased to 25 °C (0.34 °C/min) for 1 h.

### 2.3. Particle Size Distribution

#### 2.3.1. Geometric Particle Size Distribution by Laser Diffraction Measurement (LD)

LD measurement was performed by particle size analyzer (Microtrac Bell, Osaka, Japan) for sprayed sample. In this measuring system, samples are sprayed into the measuring space and irradiated with laser light. The particle size distribution is measured by detection of the scattered light.

A part of the freeze-dried cake was picked up with a micro spoon and sprayed by impacting compressed air (0.35 MPa; equivalent to flow rate at approximate 4000 L/min) using a dry dispersion apparatus PD-10S (Microtrac Bell). The scattered laser was detected through the lens of 300 mm and the particle size distribution was then measured every 0.02 ms for 2 s using a particle size distribution measuring apparatus LDSA-3500A (Microtrac Bell). In the cumulative particle size distribution, the percentage of fine particles of 5 µm or less was defined as LD FPF%_≤5 μm_, and the geometric particle size of the cumulative percentage of 50% was defined as D_50_. Each sample was measured in triplicate.

#### 2.3.2. Aerodynamic Particle Size Distribution by Aerodynamic Particle Sizer (APS) Spectrometer

Particles are drawn into the aerosol diluter (TSI, Shoreview, MN, USA) of the APS at a flow rate of 5 L/min and then diluted in the conventional APS system. Diluted particles are introduced into the APS spectrometer (TSI, Shoreview, MN, USA) at same flow rate of 5 L/min to measure the aerodynamic particle size distribution. As just described, it is not possible to measure the aerodynamic particle size distribution by APS under conditions reflecting human inhalation as opposed to MSLI measurement. However, to obtain the result of APS measurement from which the results of MSLI measurement can be estimated, the size distribution of particles introduced into the APS needs to be measured under the same condition as for MSLI measurement, especially when particles generated from breath-actuated dry powder inhalers are measured, because breath-actuated dry powder inhalers such as LDPI used in this study are aerosolized by the patient’s inspiration. Thus, we added attachments for dispersion to the APS shown in Figure 1 so that particles measured by APS can be dispersed from formulations under the same condition as for MSLI measurement (flow rate of 30 L/min at a pressure drop of 4 kPa). In this APS system, LDPI formulations are aerosolized at a flow rate of 30 L/min with a two-way needle device (Otsuka, Tokyo, Japan) (Figure 2) given a pressure drop of 4 kPa, and then the particles are introduced into the APS at a flow rate of 5 L/min. The other flow is recovered at a rate of 25 L/min by vacuum pump. The main features of the APS with dispersion attachments are that formulations are aerosolized under the same condition as for MSLI measurement, which reflects human inhalation, and that the size distribution of the generated particles can be measured quickly by APS (Figure S1).

To set the dispersion condition for APS measurement to match that for MSLI measurement, a custom-made glass throat was placed on top of the aerosol diluter of the APS (flow rate 5 L/min; Model 3302A, TSI, Shoreview, MN, USA), and a linear vacuum pump (flow rate adjusted to 25 L/min; VP0940, Nitto Kohki, Tokyo, Japan) was added (Figure 1). The freeze-dried cake was micronized under the above conditions. The particle size distribution was measured every 1 s for 8 s with an APS spectrometer (Model 3321, TSI). In cumulative particle size distribution, the percentage of fine particles of 5 µm or less was defined as APS FPF%_≤5 μm_. Each sample was measured in triplicate.

#### 2.3.3. Aerodynamic Particle Size Distribution by MSLI

The measurement by MSLI was conducted at a flow rate of 30 ± 0.3 L/min with a two-way needle device (Figure 2) given a pressure drop of 4 kPa. A vacuum pump (HCP5), critical flow controller (TPK2000), and flow meter (DFM2000) (all from Copley Scientific Limited, Nottingham, UK) were used to maintain the above condition. A hydrophilic poly(vinylidene fluoride) membrane with a diameter of 90 mm and a retention diameter of 0.65 μm (Merck Millipore, Burlington, MA, USA) was placed at stage 5 of the MSLI. Under the above condition, the freeze-dried cake was dispersed into aerosols for 8 s. Each deposition experiment was repeated in triplicate.

The diluent (hGhrelin; 0.01% benzalkonium chloride in 10 mM acetic acid, VB_3_ and Phe; purified water, VB_12_; 0.01% aqueous solution of benzalkonium chloride, Am80; 0.1% aqueous solution of Tween80/DMSO mixture (4:1)) at each stage of the MSLI was removed for analysis. The vial, device, induction port, and membrane were each washed with the diluent. HPLC (Prominence series, Shimadzu, Kyoto, Japan) was used to quantify the fraction of main drugs recovered from the vial, the device, the induction port, and each of Stages 1–5 of the MSLI. Sample solution was applied in a column at a temperature of 40 °C with a flow rate of 1.0 mL/min. Main drugs were detected by UV absorption photometer. For quantification of hGhrelin, UV absorption at wavelength 214 nm was detected, and the HPLC system was equipped with a TSKgel ODS-80Ts (150 × 4.6 mm, 3 µm; TOHSO, Tokyo, Japan) column. The concentration gradient of the mobile phase was controlled as follows: from 90% mobile phase A (0.1% aqueous solution of TFA) and 10% mobile phase B (0.085% TFA in acetonitrile); 10 min, 24% mobile phase B; 25 min, 30% mobile phase B; 26–29 min, 70% mobile phase B. For quantification of VB_3_ and Phe, UV absorption at wavelength 220 nm was detected, and the HPLC system was equipped with a PEGASIL ODS (150 × 4.6 mm, 5 µm; Senshu Scientific, Tokyo, Japan) column. The concentration gradient of the mobile phase (1% aqueous solution of ammonium acetate and methanol) was controlled as follows: 0–2 min, 1% methanol; 20–25 min, 25% methanol. For quantification of VB_12_, UV absorption at wavelength 550 nm was detected, and the HPLC system was equipped with an L-column ODS (250 × 4.6 mm, 5 µm; CERI, Tokyo, Japan) column. The concentration gradient of the mobile phase (water and acetonitrile) was controlled as follows: 0–2.5 min, 5% acetonitrile; 10–17.5 min, 20% acetonitrile. For quantification of Am80, UV absorption at wavelength 285 nm was detected, and the HPLC system was equipped with an L-column ODS (250 × 4.6 mm, 5 µm; CERI) column. The concentration gradient of the mobile phase (1% aqueous solution of ammonium acetate and acetonitrile) was controlled as follows: 0–1.5 min, 25% acetonitrile; 12.5–20 min, 50% acetonitrile.

The fine particle fraction MSLI FPF%_≤5 μm_ was defined as the ratio of the main drug of 5 μm or less to the emission. The proportion of the main drug of 5 μm or less was calculated by interpolation of a linear regression line from a plot of cumulative mass proportion of the main drug deposited on the stage (Stages 3–5) vs. logarithmic effective cut-off diameter of the respective stages (at a flow rate of 30 L/min; effective cut-off diameters for Stages 2–4 were 9.6, 4.4, and 2.4 μm, respectively). The emission was defined as the mass ratio of the emitted dose to input mass of the main drug. The emitted dose was the sum of the main drug mass of the induction port and each of Stages 1–5 of the MSLI. Each assayed mass relative to the input mass of the main drug was as follows; hGhrelin 101.2 ± 4.0%, VB_3_ 90.3 ± 2.6%, VB_12_ 90.9 ± 1.8%, Am80 99.7 ± 1.8%, and Phe 93.8 ± 0.6% (mean ± S.D., *n* = 3).

In cumulative particle distribution, the aerodynamic particle size of the cumulative percentage of 50% was defined as MMAD (mass median aerodynamic diameter).

### 2.4. Statistical Analysis

Excel (version Office 365 MSO) was used for statistical analysis. LD FPF%_≤5 μm_ or APS FPF%_≤5 μm_ was plotted against MSLI FPF%_≤5 μm_. Pearson’s correlation coefficient (r) was calculated and tested setting the critical *p*-value as 0.05 (*t*-test, the null hypothesis: There is no correlation).

## 3. Results

### 3.1. Correlation between MSLI and LD Measurement

**Figure 3:**
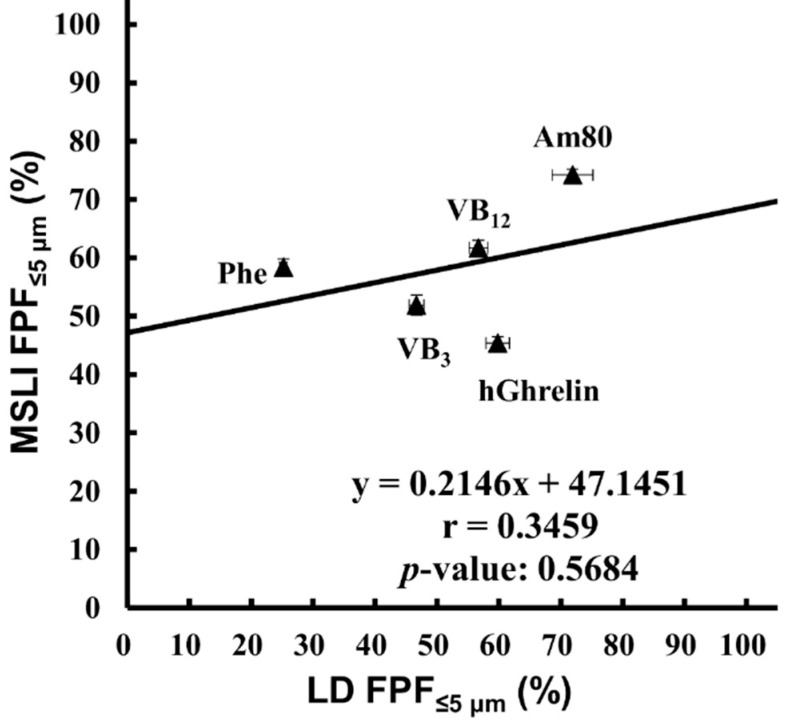
Correlation between MSLI FPF%_≤5 μm_ and LD FPF%_≤5 μm_. Values of LD FPF%_≤5 μm_ (mean ± S.E., *n* = 3) were plotted against values of MSLI FPF%_≤5 μm_ (mean ± S.E., *n* = 3). MSLI, multi-stage liquid impinger; FPF, fine particle fraction; LD, laser diffraction; hGhrelin, hGhrelin formulation containing 0.1 mg/vial of hGhrelin and 0.5 mg/vial of Phe; VB_3,_ VB_3_ formulation containing 0.1 mg/vial of VB_3_ and 0.5 mg/vial of Phe; VB_12,_ VB_12_ formulation containing 0.1 mg/vial of VB_12_ and 0.6 mg/vial of Phe; Am80, Am80 formulation containing 0.1 mg/vial of Am80 and 0.1 mg/vial of Phe; Phe, placebo formulation containing 0.2 mg/vial of Phe.

LD is widely used as a method of measuring the particle size. This method is easy to perform and enables rapid measurement; however, it measures the geometric particle size. It is not possible to directly measure the aerodynamic particle size by a laser diffraction method [34,35]. The measurement of the aerodynamic particle size distribution by MSLI and that of the geometric particle size distribution by LD were carried out using the model formulations (hGhrelin formulation containing 0.1 mg/vial of hGhrelin and 0.5 mg/vial of Phe, VB_3_ formulation containing 0.1 mg/vial of VB_3_ and 0.5 mg/vial of Phe, VB_12_ formulation containing 0.1 mg/vial of VB_12_ and 0.6 mg/vial of Phe, Am80 formulation containing 0.1 mg/vial of Am80 and 0.1 mg/vial of Phe, and placebo formulation containing 0.2 mg/vial of Phe). The comparison of FPF%_≤5 μm_ (Figure 3) resulted in r = 0.3459 with a *p*-value of 0.5684.

### 3.2. Correlation between MSLI and APS Measurement

**Figure 4:**
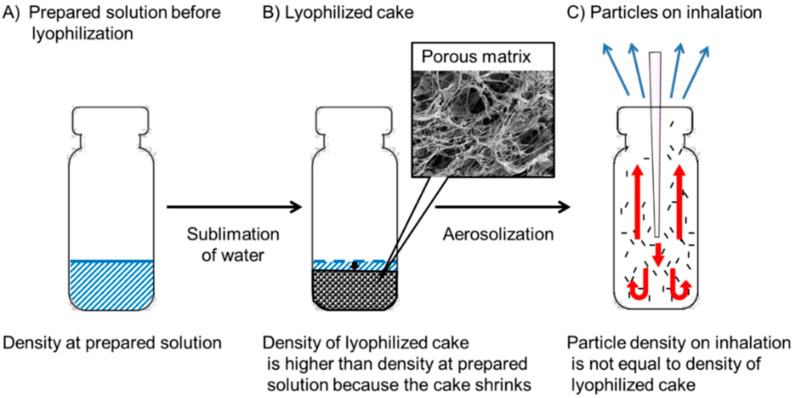
The density at each stage of lyophilizate for dry powder inhalation: (**A**) Stage 1, prepared solution before lyophilization; (**B**) Stage 2, lyophilized cake; and (**C**) Stage 3, particles on inhalation.

The particle density needs to be considered before APS measurement. LDPI acts as an inhalation system through three stages (Figure 4): Stage 1, prepared solution before lyophilization; Stage 2, sublimation of water followed by formation of a lyophilized cake; and Stage 3, disintegration of the lyophilized cake and generation of fine particles by air impact. In Stage 1, assuming that an ideal lyophilized cake is formed in which solvents are completely removed without any change in volume or shape from the prepared solution filled in the vial, the density of the lyophilized cake is the value calculated as the amount of solute divided by the volume of the prepared solution filled in the vial. It is approximated to the mass volume concentration. Thus, the value calculated as the mass (g) of the solute in the prepared solution divided by the amount (mL) of the prepared solution filling the vial is defined as the density at prepared solution (Figure 4A). In reality, a lyophilized cake would be never formed without any change in volume, and the lyophilized cake shrinks slightly, especially when the prepared solution has a low concentration as in the model formulations used in this study [36,37]. In Stage 2, because of sublimation of water, the volume of the lyophilized cake decreases from the filling volume of the prepared solution before lyophilization although the decrease in volume is not constant. Sublimation of water also forms pores randomly in the porous matrix. Thus, the density of the lyophilized cake (Figure 4B) is higher than the density at prepared solution before lyophilization (Figure 4A) and is difficult to estimate accurately. Next, in Stage 3, upon inhalation, the impact of air disintegrates the lyophilized cake and increases its porosity. Then, cake fragments are reconstructed into porous particles with further change in density. LDPI formulations are prepared as lyophilizate, whereas other porous DPIs are prepared as particles; hence, the particle density on inhalation of LDPI cannot be measured from the formulations. LDPI is aerosolized just on inhalation. The lyophilizate of the porous matrix is broken into pieces by air impact, and then these pieces are reconstructed into porous particles that are emitted through the LDPI device. The particle density on inhalation (Figure 4C) is not equal to the density of the lyophilized cake (Figure 4B) because fine particles are generated just on inhalation in LDPI system as described above.

To obtain the particle density on inhalation of LDPI, the particle density was calculated using MMAD and the D_50_ obtained from MSLI measurement and from LD measurement, respectively. The particle density on inhalation should be calculated by Equation (1). However, the dynamic shape factor (*χ*) of particles generated on LDPI cannot be determined because the lyophilized cake is instantaneously aerosolized on inhalation. Hence, the particle density of LDPI was calculated by Equation (2) [38]. The particle density calculated by Equation (2) is shown in Table 1.

(2) $$\mathrm{MMAD}=D_{50}\times \sqrt{\mathrm{Particle}\;\mathrm{Density}}$$

**Figure 5:**
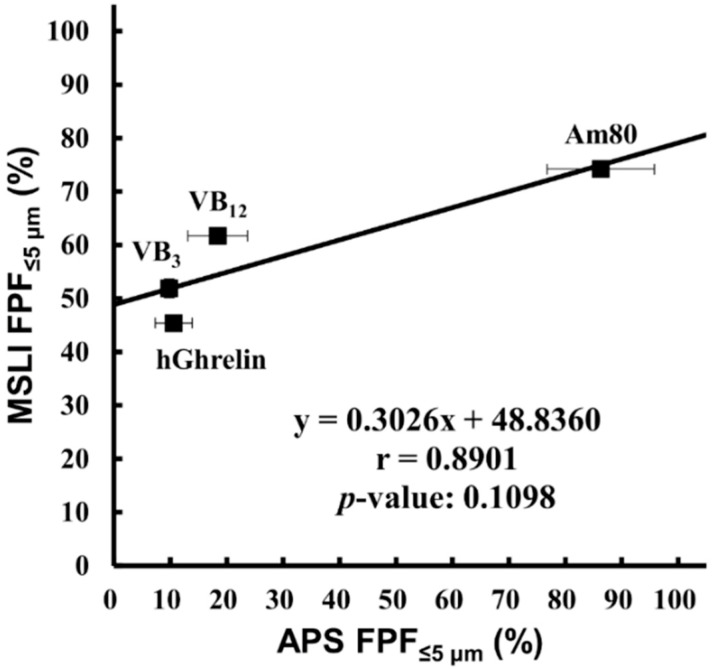
Correlation between MSLI FPF%_≤5 μm_ and APS FPF%_≤5 μm_ using the particle density calculated from MMAD and D_50_. Values of APS FPF%_≤5 μm_ using the particle density described in Table 1 (mean ± S.E., *n* = 3) were plotted against values of MSLI FPF%_≤5 μm_ (mean ± S.E., *n* = 3). MSLI, multi-stage liquid impinger; FPF, fine particle fraction; APS, aerodynamic particle sizer; MMAD, mass median aerodynamic diameter; hGhrelin, hGhrelin formulation containing 0.1 mg/vial of hGhrelin and 0.5 mg/vial of Phe; VB_3_, VB_3_ formulation containing 0.1 mg/vial of VB_3_ and 0.5 mg/vial of Phe; VB_12_, VB_12_ formulation containing 0.1 mg/vial of VB_12_ and 0.6 mg/vial of Phe; Am80, Am80 formulation containing 0.1 mg/vial of Am80 and 0.1 mg/vial of Phe.

APS FPF%_≤5 μm_ values using the particle density calculated by Equation (2) were compared with MSLI FPF%_≤5 μm_ values (Figure 5), with the result that the FPF%_≤5 μm_ showed a strong positive correlation (r = 0.8901) but no significance (*p* = 0.1098).

**Figure 6:**
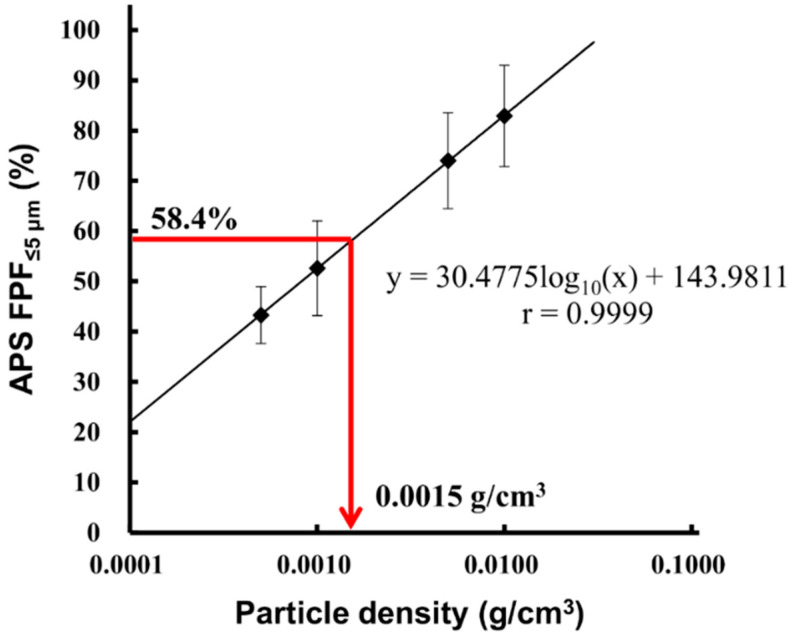
Particle density on inhalation of placebo (Phe 0.2 mg/vial). APS FPF%_≤5 μm_ of the placebo was calculated with the particle density changed in the range of 0.0005–0.01 g/cm^3^ (mean ± S.E., *n* = 3) and plotted against the particle density. The particle density at which APS FPF%_≤5 μm_ value equaled that of MSLI FPF%_≤5 μm_ (58.4%) was calculated as 0.0015 g/cm^3^. Red arrow showed the particle density when APS FPF%_≤5 μm_ value was 58.4%. APS, aerodynamic particle sizer; FPF, fine particle fraction; MSLI, multi-stage liquid impinger.

Next, APS and MSLI measurement of LDPI containing 0.2 mg/vial of Phe was performed, and APS FPF%_≤5 μm_ was calculated with the particle density changed in the range of 0.0005–0.01 g/cm^3^. The particle density at which APS FPF%_≤5 μm_ value equaled that of MSLI FPF%_≤5 μm_ (58.4%) was calculated by interpolation from a plot of APS FPF%_≤5 μm_ vs. particle density. As shown in Figure 6, the particle density of LDPI containing 0.2 mg/vial of Phe was 0.0015 g/cm^3^.

Subsequently, we tried to calculate the particle density for APS measurement from the density at prepared solution. The particle density of placebo (Phe 0.2 mg/vial), 0.0015 g/cm^3^, is the value obtained by multiplying the density at prepared solution 0.0004 by 3.75. Thus, 3.75 was set as the conversion factor, and the particle density on inhalation was taken as the particle density calculated by Equation (3). The particle density on inhalation of each formulation as calculated by Equation (3) is shown in Table 2.

Particle density on inhalation = The density at prepared solution × Conversion factor (3.75)(3)

**Figure 7:**
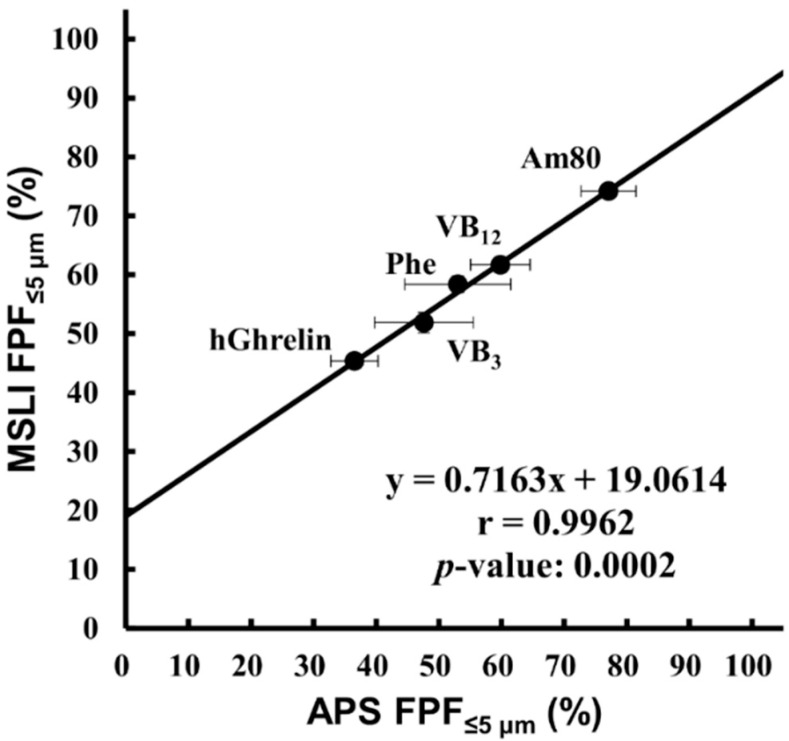
Correlation between MSLI FPF%_≤5 μm_ and APS FPF%_≤5 μm_ using the particle density calculated by the conversion factor. Values of APS FPF%_≤5 μm_ using the particle density described in Table 2 (mean ± S.E., *n* = 3) were plotted against values of MSLI FPF%_≤5 μm_ (mean ± S.E., *n* = 3). MSLI, multi-stage liquid impinger; FPF, fine particle fraction; APS, aerodynamic particle sizer; hGhrelin, hGhrelin formulation containing 0.1 mg/vial of hGhrelin and 0.5 mg/vial of Phe; VB_3,_ VB_3_ formulation containing 0.1 mg/vial of VB_3_ and 0.5 mg/vial of Phe; VB_12,_ VB_12_ formulation containing 0.1 mg/vial of VB_12_ and 0.6 mg/vial of Phe; Am80, Am80 formulation containing 0.1 mg/vial of Am80 and 0.1 mg/vial of Phe; Phe, placebo formulation containing 0.2 mg/vial of Phe.

APS FPF%_≤5 μm_ obtained by setting the particle density on inhalation calculated by Equation (3) as the density was compared with MSLI FPF%_≤5 μm_ (Figure 7). The result showed that there was a significant correlation (r = 0.9962, *p* = 0.0002).

## 4. Discussion

### 4.1. Correlation between MSLI and LD Measurement

LD is a method to measure the geometric particle size distribution. The geometric particle size does not depend on the particle density. In contrast, MSLI is a method to measure the aerodynamic particle size distribution. Dispersion condition is also different between LD and MSLI. In LD measurement, a part of the lyophilized cake of LDPI formulation is dispersed by compressed air (0.35 MPa; equivalent to flow rate at approximate 4000 L/min). On the other hand, in MSLI measurement, the whole lyophilized cake of LDPI formulation in a vial is dispersed though the inhalation device under the condition reflecting human inhalation (flow rate of 30 L/min at a pressure drop of 4 kPa). Due to the difference of dispersion condition, it was considered that particles measured by LD were different in size and shape from particles upon inhalation.

Form the above, the results of LD were compared with that of MSLI to confirm that there was no correlation, although LD is a widely used method. In practice, there was no significant correlations between LD and MSLI as expected (Figure 3).

### 4.2. Correlation between MSLI and APS Measurement

The aerodynamic particle size distribution is measured by APS based on TOF theory. The conventional APS differs from MSLI in the dispersion condition and measurement theory. However, regarding the APS with dispersion attachments used in this study, the dispersion condition is the same as that for MSLI measurement (Figure S1). In this APS system with dispersion attachments, the whole lyophilized cake of LDPI formulation in a vial was used for measurement as well as MSLI. The sample for measurement was not picked from the formulation, which was the sampling method for LD measurement. However, the size distribution of particles generated from the whole lyophilized cake cannot be measured by APS if particles are introduced into the APS directly, because the particle concentration is too high to measure the particle size distribution by TOF. Thus, in the APS system used in this study, particles are diluted in the following two steps and then introduced into the APS at standard flow rate for APS measurement (5 L/min). In the first step, particles are generated under the same condition as for MSLI which reflects human inhalation (flow rate of 30 L/min at a pressure drop of 4 kPa) and then introduced at flow rate of 30 L/min into the glass throat which is equivalent to the induction port of MSLI. At the flow of 30 L/min, the flow necessary for standard APS measurement is 5 L/min. The other flow is 25 L/min, and particles in this flow are recovered randomly by vacuum pump. Hence, particle concentration decreases. In the second step, particles at the flow of 5 L/min are introduced into the aerosol diluter and diluted uniformly to the appropriate concentration for TOF measurement. Then, diluted particles are introduced into the APS at flow rate of 5 L/min to measure the size distribution. From the above, the aerodynamic particle size distribution of same particles is measured by MSLI or APS. Thus, we examined the correlation between MSLI and APS focusing on the particle density.

When using the APS, attention must be paid to the influence of the particle density value on the results, the main problem of which has been the measurement of aerodynamic particle size distribution in pressurized metered-dose inhalers [24,39]. The particle density value is necessary to perform APS measurement, and its setting has a large effect on the results of APS measurement (Figure 6). However, it is difficult to obtain accurate particle density of DPIs formulated with large porous particles because they behave as agglomerates of primary particles. Pores of porous particles can either be opened pores (connected to other pores to the surface or not) or closed pores in particles. Because the size, shape, or orientation of the pores and the volume occupied by the pores change due to aggregation, it is difficult to estimate the density of porous particles accurately [40,41]. Furthermore, it is much more difficult to obtain accurate particle density of LDPI in which lyophilizate pieces behave as porous particles. In the LDPI system, the lyophilizate of the porous matrix with a fiber-like network structure is broken into pieces by air impact, and then these pieces are reconstructed into porous particles suitable for pulmonary administration [27,28,42]. Because porous particles of LDPI are generated just on inhalation, their porosity cannot be measured accurately. Thus, accurate particle density of LDPI is difficult to obtain, but this problem can be solved by using a conversion factor. The particle density on inhalation can be calculated by the conversion factor based on the results of MSLI measurement. The conversion factor was obtained from the particle density at which APS FPF%_≤5 μm_ value equaled that of MSLI FPF%_≤5 μm_. This means that the particle density on inhalation can be determined by setting the particle density value for APS measurement under the same condition as MSLI so that the APS FPF%_≤5 μm_ value equals that of MSLI FPF%_≤5 μm_. In practice, a significant correlation between MSLI FPF%_≤5 μm_ and APS FPF%_≤5 μm_ was obtained by setting the particle density on inhalation calculated by the conversion factor (Equation (3)) as the particle density (Figure 7). This indicates that, in LDPI systems, the results of MSLI measurement can be estimated from APS measurement using the particle density determined by Equation (3).

In the present study, the conversion factor was calculated as 3.75. The conversion factor of 3.75 could be applied to five types of formulations with different compounds and different contents, even though the physical properties of the five compounds differed obviously. This showed that the conversion factor does not depend on the type and content of the compound. On the other hand, formulations were prepared by filling the same volume of the prepared solution using vials of the same size. Because LDPI is aerosolized by air impact, the convection flow of air in the vial is essential for aerosolization. Therefore, it is considered that the conversion factor for LDPI becomes a different value when the space in the vial for air convection changes due to a change in the size of the vial or the filling volume. In other words, if formulations are prepared by filling the same volume using vials of the same size, the result of APS that closely agrees to the result of MSLI can be obtained by using conversion factor of 3.75 regardless of the type or content of compounds. Even if the size of the vial or the filling volume is changed, the conversion factor can be calculated by the same way as in this study. At the same time, in LDPI, the particle density on inhalation depends on the concentration of the prepared solution (Equation (3)). LDPI acts as an inhalation system through three stages (Figure 4), and the change in volume and density occurs more than once. The density change in LDPI is complicated, but the rate of density change from the prepared solution to the particles on inhalation is considered to be constant (=3.75 times) from Equation (3).

In addition, we believe that the aerodynamic particle size distribution of DPIs formulated with large porous particles other than LDPIs can be measured in the same way as LDPI in this study for the following reasons. First, the APS with dispersion attachments can disperse formulations under the same condition for MSLI measurement, which reflects human inhalation. Second, the density of porous particles can be estimated by using the conversion factor, even though it is difficult to measure the density of the porous particles directly. The conversion factor is determined by calculating the density so that the value obtained from the APS equals that from MSLI. Therefore, we expect our simple method using an APS with dispersion attachments and the conversion factor to be useful in easily and quickly measuring the aerodynamic particle size distribution of porous particles.

It is also the point to be considered that APS measurement based on the TOF theory cannot measure particles of the main drug separately from particles of other components [39]. If lyophilized cake is aerosolized into particles for which the size differs between main drugs and excipients, the result of APS measurement, even corrected in the particle density, will not agree with the result of MSLI measurement. In practice, there was a strong and significant correlation between MSLI FPF%_≤5 μm_ and APS FPF%_≤5 μm_ obtained by setting the particle density on inhalation calculated by the conversion factor as the particle density when five compounds of different physical properties were used (Figure 7). This means that main drugs and excipients are in the same particles generated from lyophilized cake by air impact. Thus, particles measured by APS were considered to be particles of the main drug in LDPI.

## 5. Conclusions

There was a significant correlation between results of MSLI and that of the APS with dispersion attachments and the conversion factor. In the APS with dispersion attachments, formulations are aerosolized under the same condition as for MSLI measurement, while the size distribution of the generated particles can be measured in a much shorter time than MSLI. The conversion factor made it possible to calculate the density of porous particle generated on inhalation of LDPI. Thus, by using the APS with dispersion attachments setting particle density calculated by the conversion factor as the particle density value, the aerodynamic particle size distribution of many samples can be measured in a short time and the result of MSLI can be estimated from the obtained result.

## Figures and Tables

**Table 1:** Particle density calculated from MMAD and D_50_.

Drug or Compound	MMAD (µm)	D_50_ (µm)	Particle Density (g/cm^3^)
hGhrelin	4.85	4.01	1.4628
VB_3_	4.19	5.95	0.4959
VB_12_	3.93	4.14	0.9011
Am80	2.98	3.55	0.7047

MMAD, mass median aerodynamic diameter.

**Table 2:** Particle density calculated by conversion factor (3.75).

Drug or Compound	Density at Prepared Solution	Particle Density on Inhalation (g/cm^3^)
hGhrelin	0.00120	0.00450
VB_3_	0.00120	0.00450
VB_12_	0.00140	0.00525
Am80	0.00040	0.00150
Placebo (Phe 0.2 mg/vial)	0.00040	0.00150

## Supplementary Materials

The following are available online at https://www.mdpi.com/1999-4923/12/10/976/s1, Figure S1: Multi-stage liquid impinger and aerodynamic particle sizer (APS) system with dispersion attachments, Table S1: Stock solutions used for preparation of freeze-dried cake, Video S1: High speed camera records of aerosolization of LDPI.
