# Supplementary material for: Simple Method to Measure the Aerodynamic Size Distribution of Porous Particles Generated on Lyophilizate for Dry Powder Inhalation
Source: Pharmaceutics. 2020 Oct 15;12(10):976. doi: 10.3390/pharmaceutics12100976 (PMC7650659; doi:10.3390/pharmaceutics12100976)
Supplement: Supplementary file 1 [file pharmaceutics-12-00976-s001.zip › Supplementary material/Supplementary materials.docx]

Simple Method to Measure the Aerodynamic Size Distribution of Porous Particles Generated on Lyophilizate for Dry Powder Inhalation

Kahori Miyamoto, Hiroaki Taga, Tomomi Akita and Chikamasa Yamashita

**
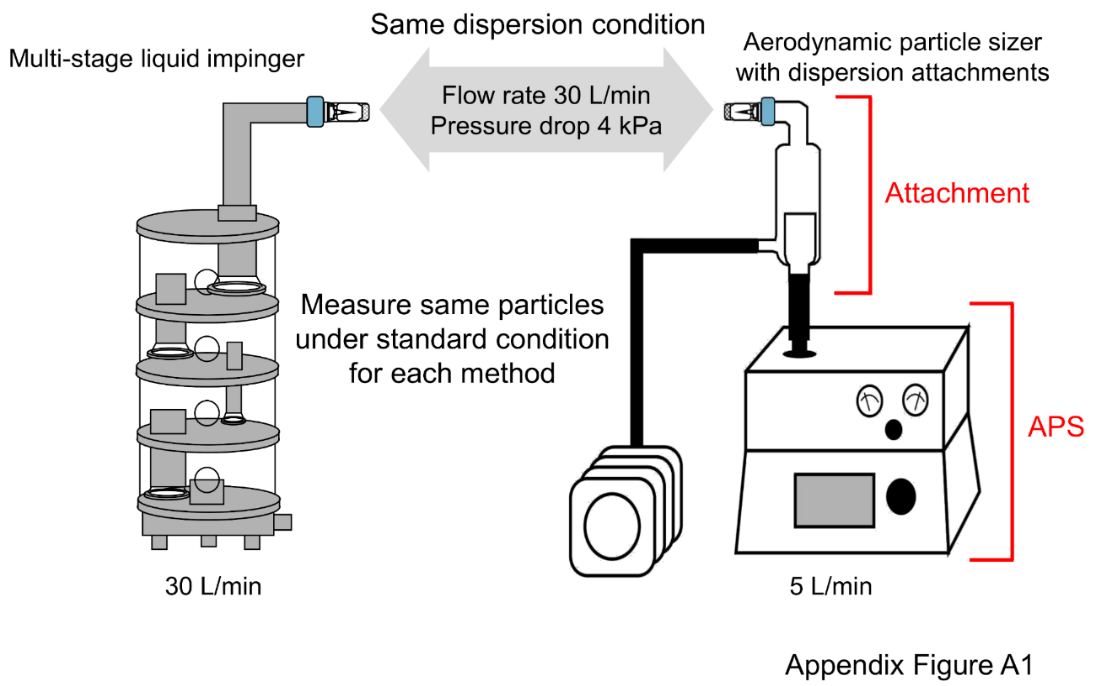
**

**Figure S1.** Multi-stage liquid impinger and aerodynamic particle sizer (APS) system with dispersion attachments. Formulations are dispersed under the same condition as for multi-stage liquid impinger measurement (flow rate of 30 L/min at a pressure drop of 4 kPa with two-way needle device). Dispersed particles are measured under the standard condition for each method. In MSLI, the aerodynamic particle size distribution is measured at flow rate of 30 L/min by mass. In APS, the aerodynamic particle size distribution is measured at flow rate of 5 L/min based on time-of-flight theory.

**Table S1.** Stock solutions used for preparation of freeze-dried cake.

| **Drug or Compound** | **Stock Solution (mg/mL)** | **Solvent** |
| --- | --- | --- |
| hGhrelin | 2 | Purified water |
| VB_3_ | 2 | Purified water |
| VB_12_ | 2 | Purified water |
| Am80 | 20 | Ethanol (99.5%) |
| Phe | 20 | Purified water |

**Video S1**. High speed camera records of aerosolization of LDPI. The air introduced in synchronization with the patient’s inspiration convects in the vial similar to air in a jet mill. The lyophilized cake is broken into pieces by the convention flow of air, and these pieces are reconstructed into particles while convecting in the vial with air flow. These reconstructed particles are emitted from the vial.
